# Conventional vs. Tablet Computer-Based Patient Education following Lung Transplantation – A Randomized Controlled Trial

**DOI:** 10.1371/journal.pone.0090828

**Published:** 2014-03-07

**Authors:** Hendrik Suhling, Jessica Rademacher, Imke Zinowsky, Jan Fuge, Mark Greer, Gregor Warnecke, Jacqueline M. Smits, Anna Bertram, Axel Haverich, Tobias Welte, Jens Gottlieb

**Affiliations:** 1 Dept. of Respiratory Medicine, Hannover Medical School, Hannover, Germany; 2 Dept. of Cardiothoracic, Transplantation and Vascular Surgery, Hannover Medical School, Hannover, Germany; 3 Eurotransplant International Foundation, Leiden, The Netherlands; 4 Dept. of Nephrology and Hypertension, Hannover Medical School, Hannover, Germany; University of California Los Angeles, United States of America

## Abstract

**Background:**

Accurate immunosuppression is of critical importance in preventing rejection, while avoiding toxicity following lung transplantation. The mainstay immunosuppressants are calcineurin inhibitors, which require regular monitoring due to interactions with other medications and diet. Adherence to immunosuppression and patient knowledge is vital and can be improved through patient education. Education using tablet-computers was investigated.

**Objective:**

To compare tablet-PC education and conventional education in improving immunosuppression trough levels in target range 6 months after a single education. Secondary parameters were ratio of immunosuppression level measurements divided by per protocol recommended measurements, time and patient satisfaction regarding education.

**Design:**

Single-centre, open labelled randomised controlled trial.

**Participants:**

Patients >6 months after lung-transplantation with <50% of calcineurin inhibitor trough levels in target range.

**Intervention:**

Tablet-pc education versus personal, nurse-led education.

**Measurements:**

Calcineurin inhibitor levels in target range 6 months after education, level variability, interval adherence, knowledge and adherence was studied. As outcome parameter, renal function was measured and adverse events registered.

**Results:**

Sixty-four patients were 1:1 randomised for either intervention. Levels of immunosuppression 6 months after education were equal (tablet-PC 58% vs. conventional 48%, p = 0.27), both groups improved in achieving a CNI trough level within target range by either education method (delta tablet-PC 29% vs. conventional 20%). In all patients, level variability decreased (−20.4%), whereas interval adherence remained unchanged. Knowledge about immunosuppression improved by 7% and compliance tests demonstrated universal improvements with no significant difference between groups.

**Conclusion:**

Education is a simple, effective tool in improving adherence to immunosuppression. Tablet-PC education was non-inferior to conventional education.

**Trial Registration:**

ClinicalTrials.gov NCT01398488 http://clinicaltrials.gov/ct2/show/NCT01398488?term=gottlieb+tablet+pc+education&rank=1.

## Introduction

It has been previously demonstrated in a variety of chronic diseases that non-adherence to medication and other forms of treatment is a major problem [1], which may impact on long-term outcomes [2], [3]. Numerous reasons for non-adherence have been reported, including insufficient information, anxiety of side-effects, treatment cost, forgetfulness and lack of perceived benefit [4]. Patient education and awareness is considered pivotal in improving adherence, with various concepts having already been developed to address this [5], [6]. Patient educational needs vary greatly depending on their underlying condition, with diseases demanding precise medication dosing (diabetes mellitus) or modifications in health-related behaviour (COPD) appearing to profit most from educational programs [7]–[10].

Following organ transplantation, patients require highly complex treatment regimes based on various immunosuppressant drugs that have small therapeutic ranges and profound side-effect profiles [11]. Sub-therapeutic immunosuppression remains a leading cause of allograft rejection, graft loss, and death [12]. Indirectly it is associated with decreased quality of life and inevitably increased health care costs. Previous studies have demonstrated non-adherence rates in calcineurin inhibitors (CNI) ranging between 13 and 22% [13]. Non-adherence increases over time after transplantation [13], [14].

Conventional patient-education requires a trained specialist, a suitable location and is time-intensive [15]. Computer-based patient education has been attempted, with reports suggesting that it can provide a more cost-effective method of educating patients [16]. Tablet-PCs, with their user-friendly interfaces and large screens improve simplicity and can be handled even by chronically ill or elderly patients. This study investigated whether tablet-PC education could improve immunosuppression adherence amongst lung transplant recipients compared to conventional education strategies.

## Materials and Methods

### Study design and patient collective

A prospective randomized open labelled control trial was undertaken at a single university centre (Hannover Medical School, Germany), comparing tablet–PC to conventional patient education. Patient recruitment occurred between August 2011 and July 2012. After inclusion, patients first answered a questionnaire assessing their understanding of the various important aspects related to CNI treatment (further described below), before being randomized 1:1 into either of the 2 education groups. At the same visit, patients then participated either in self-directed tablet-PC education or were counselled by a trained nurse (I.Z). Both education content was identical. Six months later they completed the initial questionnaire for a second time.

**Figure 1:**
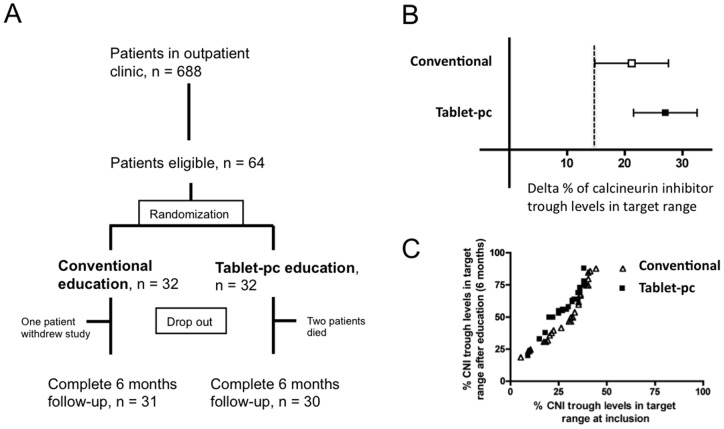
Flow chart of inclusion and improvement of immunosuppression. Flow chart of inclusion (**A**). Delta % of calcineurin inhibitor trough levels in target range 6 months after patient education compared to 6 months before patient education (**B**). Dashed line marks cut-off of non-inferiority (lower 95% CI of conventional group, p = 0.17). Visualization of calcineurin inhibitor levels at inclusion (x-axis) and after 6 months (y-axis) (**C**).

Follow-up was 6 months after start of the education (Figure 1).

All patients provided written informed consent. The study was approved by the Internal Review Board of the Hannover Medical School (No. 1019–2011) and registered under clinicaltrials.gov, No. NCT01398488.

### Inclusion and exclusion criteria

All patients aged ≥18 years, who had undergone a single, double or heart-lung-transplantation ≥6 months and who regularly participated in our post-transplantation surveillance program were screened for eligibility. Our program provides exclusive centralized monitoring of calcineurin inhibitors (CNI) for all patients at our central lab. Local physicians mail patient blood samples at specified intervals for analysis. To qualify for study participation, patients required a minimum of 10 CNI trough levels in the preceding 6 months, of which less than 50% were in the target range. Patients who were hospitalized during the previous 3 months, who had advanced chronic lung allograft dysfunction (stage 3), chronic kidney disease K/DOQI stage V (eGFR <15 ml/min/1.73 m^2^), oxygen requirement at rest or pulsed steroids in the previous 4 weeks (>500 mg methylprednisolone per day) were excluded. Illiteracy, limited German language skills, need for isolation (multi- or pan-resistant organisms) or other factors limiting patient communication or computer handling were considered additional exclusion criteria.

### Immunosuppression

Standard maintenance immunosuppression consisted of a triple drug regimen including CNI, prednisolone and mycophenolate mofetil [17]. Ciclosporine A (CSA) was the 1^st^ line CNI, with exception of combined-organ recipients who received tacrolimus. Patients with recurrent or steroid-resistant rejection episodes or CSA intolerance were switched to tacrolimus. Target CSA trough levels, as measured by liquid chromatography were 180 ng/ml (0–6 months), 140 ng/ml (6–12 months), 100 ng/ml (12–24 months) and 60 ng/ml (>24 months). A target range of ±20 ng/ml was defined. Target trough levels for tacrolimus were 12 ng/ml (0–6 months), 10 ng/ml (6–12 months), 8 ng/ml (12–24 months) and 6 ng/ml (>24 months). A target range of ±2 ng/ml was defined for tacrolimus. Patients demonstrating variable trough levels were required to send control samples every 1–2 weeks. Levels out of target range were re-checked after 1 week following dose adjustment. In stable patients, control intervals were gradually lengthened to a maximum of every 4 weeks.

### Intervention: Education materials and education content

Educational material was devised by lung-transplant specialists (J.G., H.S), and paper- and computer-based presentations of identical content were designed (J.F.). Content differed slightly depending on whether patients were receiving cyclosporine or tacrolimus, necessitating two sets of educational aids. An iPad (Apple Inc., second generation) was used in the tablet-pc group for education. A Keynote presentation (Apple Inc.) consisting of 30 slides and 4 video clips totalling 12:45 min were included. Patients unfamiliar with using an iPad received short instruction before commencing their tutorial. In the conventional group, a trained nurse-specialist (I.Z.) using the designated written material provided patient instruction.

Educational content comprised of highlighting the importance of regular medication and side-effects (e.g. rejection or infection) and subsequently provided practical tips on how to achieve stable drug levels (Table S1). Incorporated video clips emphasized evidence for immunosuppression and the importance of ongoing adherence. This included a patient explaining regular CNI intake, another illustrating correct storage of immunosuppressive drugs and one explaining common causes of variation in drug levels (Figure S1).

All patients received a single page summary sheet to take home and were encouraged to ask further questions during follow-up.

The iPads were cleaned between patients according to standard recommendations obtained from the deBac-App (available via iTunes, PLRI MedAppLab, Hannover). All software used was regularly updated to latest versions.

### Outcome measurement

Primary objective was percentage of immunosuppression levels in target range 6 months after education and the comparison between the table-pc and the conventional education group. Secondary objectives were interval adherence, which is defined as the number of measurements in which the target level is reached out of the total number of measurements and time required completing the questionnaire and documentation. The glomerular filtration rate before inclusion and at 6 months was compared and adverse events, hospitalisation, rejection or infection were monitored (Table 1).

**Table 1:** End points.

*Primary endpoint:*
Percentage of calcineurin inhibitor trough levels in target range 6 months after patient education
*Secondary endpoints:*
Improvement of percentage of calcineurin inhibitor trough levels in target range (Delta %) of the next 10 measurements after patient education compared to the last 10 measurements before patient education
Trough level variability 6 months after patient education compared to 6 months before patient education
Number of immunosuppression level measurements vs. recommended measurements
Total time of education
Total time of answering questionnaire
Improvement of patient knowledge on immunosuppressive after patient education
Patient satisfaction
Self rated adherence to immunosuppressive medication (BAASIS scale)
Therapy adherence 6 months after patient education compared to 6 months before patient education
Glomerular filtration rate 6 months after patient education compared to baseline

### Questionnaires for medication intake adherence

To assess patient medication intake adherence, all participants completed the Basel assessment of adherence with immunosuppressive medication scales (BAASIS) [18]–[20], the immunosuppressant therapy adherence barrier instrument (ITBS) [21] and the Morisky Score [22]. The BAASIS questionnaire included 4 questions (0–4 points) evaluating missed CNI consumption in the last 4 weeks, consecutive occasions were CNI medication was missed, delays of ≥2 hours in CNI consumption and autonomous CNI dose alteration. The Morisky score has been described previously (0–4 points) [22]. Higher scores in all tests correlated with better adherence. The ITBS examines 13 items using a Likert-type scale (1 =  ‘strongly disagree’ to 5 =  ‘strongly agree’) as previously described [21].

### Physicians' valuation of adherence

Physicians independently ranked patient adherence in five categories, including drug levels, physical fitness, communication with the transplantation centre, completion of daily home-spirometry and general health awareness. Good, moderate and bad adherence was differentiated.

### Questions evaluating satisfaction and knowledge

Questions relating to patient satisfaction regarding educational training and questionnaire satisfaction were also incorporated. Knowledge pertaining to immunosuppressant medication was assessed using 20 yes/no questions derived directly from the educational material. Knowledge was rated to be 0–100%.

The questionnaire was completed either electronically via tablet-PC or in written form and was provided before education and 6 months subsequently.

### Tablet-PC usage and link to local database

Patients in the tablet-PC group could use an AluPen (Just mobile, Germany) for data entry. Questionnaires were completed using FileMaker Go (v. 11, FileMaker Inc., USA) installed on the iPads, with data being transferred via WiFi in real time to a study database in FileMaker Pro 10 Server hosted on the local intranet.

### Methods against bias

All patients attending our outpatient clinic were screened for eligibility. Blinding was not undertaken. Randomisation involved an allocation sequence using numbered containers (created by J.F. using www.random.org), with patients being assigned to groups based on their inclusion number. Stratification was performed for cystic fibrosis (CF) patients due to comparatively younger age (median 27 years vs. 55 years for other diagnoses) to minimize bias due to better computer literacy among younger patients as well as their increased susceptibility to variable drug absorption that can profoundly influence drug pharmacokinetics, leading to greater fluctuation in trough levels [23].

### Statistical analysis

Our calculations indicated that a cohort of 62 patients was required to achieve statistical power of 95% in detecting a 20% difference therapeutic trough-levels between both groups. This estimation of improvement was derived from previous studies. All continuous variables are presented as median with inter-quartile ranges (25% and 75%). Likert-scales with less than 5 points (satisfaction and Morisky Score) were expressed as mean ± standard deviation. Variables were compared between the groups using student's t-test (Table 2 and 3 for numeric data) or non-parametric testing (Mann-Whitney U) (Table 4) in cases of non-normal distribution. Categorical variables were compared between the groups using the chi-square test. All reported P values are two-sided and the level of significance was set at p<0.05.

**Table 2:** Demographics.

Variable	Subgroup	All patients, n = 64	Tablet-pc group, n = 32	Conventional group, n = 32	Significance
**FEV1% baseline (%)**		93 (84; 97)	93 (82; 97)	93.5 (88.3; 96.8)	0.4
**Grade of chronic rejection (BOS grade)**	**0**	52 (82.5)	24 (78)	28 (88)	0.34
	**1**	11 (17.5)	7 (22)	4 (12)	
**Underlying disease, n (%)**	**Cystic fibrosis**	22 (34)	9 (28)	13 (41)	0.8
	**Pulmonary fibrosis**	12 (19)	7 (22)	5 (16)	
	**Emphysema**	5 (8)	2 (6)	3 (9)	
	**Pulm. Hypertension**	7 (11)	4 (12)	3 (9)	
	**other**	18 (28)	10 (32)	8 (25)	
**Transplantation, n (%)**	**Single lung-transplantation**	2 (3)	1 (3)	1 (3)	0.75
	**double lung transplantation**	62 (97)	31 (97)	31 (97)	
**Age, years**		47 (34; 57)	52 (35.9; 57.6)	45 (33.3; 53.9)	0.18
**Immunosuppression n (%)**	**Cyclosporine**	36 (56)	19 (59)	17 (53)	0.8
	**Tacrolimus**	28 (44)	13 (41)	15 (47)	
**Baseline adherence judged by physician at inclusion, n (%)**	**Good**	49 (76)	27 (84)	22 (69)	0.3
	**Moderate**	12 (19)	4 (13)	8 (25)	
	**Bad**	3 (5)	1 (3)	2 (6)	
**Adherence after 6 month) n (%)**	**Good**	47 (77)	21 (70)	26 (84)	0.4
	**Moderate**	13 (21)	8 (27)	5 (16)	
	**Bad**	1 (2)	1 (3)	0	
**Levels of immunosuppression in target range at inclusion, % (IQR)**		31 (20; 36)	31 (20.5; 36)	31 (20; 38.3)	0.77
**Absolute number of immunosuppression level within 6 months, n**	**before education**	15 (13;22)	15 (13; 22)	16 (14; 21)	0.9
	**after education**	15 (13; 19)	15 (13; 20)	14 (13; 17)	0.1
**Absolute number of immunosuppression levels in target range, n**	**before education**	5 (3; 7)	5 (3; 7)	5 (2; 7)	0.9
	**after education**	8.5 (5; 12)	10 (5.8; 14)	7 (4.8; 9.5)	0.048

Patient demographics and characteristics. Categorical variables were compared using a chi-square test and numeric values were shown as median with IQR, using student's t-test.

**Table 3:** End point results.

Variable	Time point	All patients, n = 64	Tablet-pc group, n = 32	Conventional group, n = 32	Significance
Levels of immunosuppression in target range, % (IQR)	6 months	55 (38; 68)	58 (50; 69.3)	48.5 (36; 67.3)	0.27
Improvement of percentage of calcineurin inhibitor trough levels in target range (Delta %) of the next 10 measurements after patient education compared to the last 10 measurements before patient education		20 (10; 40)	30 (10; 40)	20 (7.5; 30)	0.27
Ratio of level measurements divided by recommended measurements *	inclusion	1.11 (0.96; 1.27)	1.09 (0.90; 1.21)	1.17 (97; 1.30)	0.21
	6 months	1.14 (1.00; 1.43)	1.24 (1.07; 1.51)	1.11 (0.96; 1.28)	0.48
Improvement of percentage of calcineurin inhibitor trough levels (Delta %) in target range 6 months after patient education compared to 6 months before patient education		26 (12.5; 36)	29 (17.3; 36.3)	20 (4.8; 36)	0.17
Total time of education (first visit) (min)	inclusion	25 (21.3; 29.5)	25 (22; 28)	25 (21; 30)	0.75
Total time of answering questionnaire (first visit) (min)	inclusion	18 (14.3; 21.6)	16.5 (14; 22)	19 (16; 22)	0.38
Estimated glomerular filtration rate (% improvement 6 months to baseline)		4 (−1.2; 15.1)	4 (−1.2; 18.5)	5 (−2.5; 13.3.)	0.37

*<1: less measurements than required, >1 more measurements than required.

Results from pre-defined end-points. All values are shown as median with IQR (student's t-test).

**Table 4:** Adherence Scores.

Variable	Time point	All patients, n = 64	Tablet-pc group, n = 32	Conventional group, n = 32	Significance
**BAASIS questions ^a^**	**inclusion**	4 (3; 4)	4 (3; 4)	3 (3; 4)	0.12
	**after 6 months**	4 (4; 4)	4 (3; 4)	4 (3; 4)	0.8
**VAS ^b^**	**inclusion**	100 (96; 100)	100 (96.3; 100)	100 (96.3; 100)	
	**after 6 months**	100 (100; 100)	100 (93.8; 100)	100 (100;100)	
**ITBS Score**	**inclusion**	14 (12; 16)	14 (12; 15)	14 (12; 16)	0.56
	**after 6 months**	12 (12; 15)	13 (12; 15)	12 (12; 14)	0.8
**Morisky Score** c	**inclusion**	4 (0.29)	4 (0.25)	4 (0.34)	0.4
	**after 6 months**	4 (0.22)	4 (0.18)	4 (0.25)	0.5
**Satisfaction education ^d^**	**inclusion**	1 (0.54)	1 (0.55)	1 (0.53)	0.6
	**after 6 months**	2 (0.78)	1 (0.9)	2 (0.64)	0.27
**Satisfaction questionnaire**	**inclusion**	2 (0.77)	2 (0.55)	2 (0.91)	0.09
	**after 6 month**	2 (0.98)	2 (0.94)	2 (0.99)	0.11
**Baseline knowledge; n (%)**	**80–100%**	40 (63)	19 (59)	21 (66)	0.8
	**<80%**	24 (37)	13 (41)	11 (34)	0.8
**Knowledge %**	**inclusion**	80 (71; 90)	80 (71; 90)	85 (71; 90)	0.6
	**after 6 months**	90 (81; 95)	90 (83; 95)	90 (78; 95)	0.6
**Improvement of knowledge (%)**		7 (0; 18)	7 (0; 19)	7 (−1; 18)	0.87

Results from subjective and objective adherence (BAASIS, VAS, ITBS and Morinsky scale). ^a^ Self reported adherence: 1–4 points; 1 poor adherence, 4 very good adherence. ^b^ VAS (visual analogue scale of BAASIS questionnaire) 0 to 100; 100 very good self rated adherence.

c Mann-Whitney-U-Test, Mean (SD); ^d^ Satisfaction, 1–5 points (1 very good to 5 very bad); Mean (SD), Mann-Whitney-U-Test.

## Results

Sixty-four patients were enrolled between 5.8.2011 and 15.5.2012, with 32 patients being randomly assigned to each group. Three patients did not complete the study: two died and one withdrew from study. In total, 30 patients completed the study in the tablet-PC and 31 in the conventional group. Patient characteristics were similar between the groups (Table 2).

### Endpoint outcomes

Primary endpoint: there was no difference between the groups in regard to levels of immunosuppression in target range after 6 months (p = 0.27), see Table 3.

Following any educational intervention, significant improvements of immunosuppression levels in target range (31% to 55%; p<0.001) were observed. Absolute improvement in percentage of calcineurin inhibitor trough levels (Δ%) in target range in the 6 months before and after patient education showed no significant difference in a two-sided t-test between the groups. Overall, a 26% absolute improvement of CNI levels in target range was observed, with an interesting trend towards better performance in the tablet-PC group (20% vs. 29%, p = 0.17). Secondary end-points are displayed in Table 3.

### Knowledge and renal function

There was no difference between groups and between time-points for renal function or knowledge (Table 3 and 4).

### Self-reported and measured adherence

Results from questionnaires relating to adherence revealed no differences between inclusion and at 6 months in either group.

Three patients reported drug holidays in the preceding 4 weeks but no autonomous changes in dosage (BAASIS questionnaire). On 15 occasions prior to education, CNI intake fell outside the recommended 2-hour window, with no differences observed between groups. Following training only six patients admitted this. In ITBS, 3 patients reported that they could hardly remember taking their CNI although all knew when they should take them. Two patients reported problems correctly timed CNI dosing resulting from changes in daily schedule.

### Physicians' judgment of adherence

Physicians rated most patients as adherent at inclusion (Table 1). There was no significant improvement after 6 months (p = 0.5) and no intergroup differences were observed.

### Evaluation of tablet-PC usage

All patients participating in tablet-PC training successfully completed both the tutorial and the questionnaire. All patients rated training with the tablet-PC as good.

### Sub-group analysis

All study participants considered themselves treatment-adherent at the time of inclusion despite poor performance in achieving therapeutic trough-levels. We, therefore, examined the influence of knowledge levels at inclusion on drug level improvement (cut-off <80% knowledge corresponding to median). Low knowledge levels with regard to immunosuppression were identified in 24 patients. Analysis however revealed no significant differences in improvement in these patients compared to the remainder of the cohort (29% vs. 23% respectively; p = 0.39).

Patients rated as moderately or poorly adherent exhibited significantly lower knowledge levels (p = 0.01). Fifteen patients judged as non-adherent at inclusion displayed smaller improvements in therapeutic CNI levels compared to patients with good adherence (23% vs. 29% respectively; p = 0.38). Patients demonstrating poor existing knowledge and non-adherence (n = 10) displayed no difference in drug-level improvements based on education received (p = 0.3).

Existing level of knowledge influenced the time needed to complete the questionnaire (low knowledge: median 20 min (IQR 16–28), good knowledge: 17 min (IQR 14–20); p = 0.005), but not the duration of education (low knowledge: median 26 min (IQR 23–31), good knowledge: median 25 min (IQR 21–28); p = 0.3).

### Hospitalization, rejection and infection during follow-up

Two patients died during follow-up, with both being in the tablet-PC group. Causes of death were lymphoma and myocardial infarction. Eight patients were hospitalized during follow-up (1 tablet-PC and 7 conventional education; p = 0.05), due to progression in chronic rejection (n = 2), infection (n = 3), oesophageal biopsy (n = 1) and vascular prosthesis (n = 1). Seventeen patients suffered an infection, 9 of which were in the tablet-PC group (p = 1.0). Ten patients received pulsed steroids: 6 in the tablet-PC and 4 in the conventional group (median 123 days after inclusion, p = 0.5).

### Cost-calculation

The creative time required for tablet-PC education and questionnaire compared to that for conventional education was assumed to be equal. The initial equipment outlay for tablet-PC education included an iPad (499 €), an Apple AirPort Express (79 €), the required software (Keynote for iPad, Filemaker Pro for iPad, Filemaker Server and Client, totalling 852 €). Short instruction in using the tablet-PC was usually required (5 min, performed by study nurse). Conventional education was provided by a study nurse (approx. 50 €/h employer costs) and lasted around 30 min per patient. Taken together, the cost of each educational session in tablet-PC group was 45 €, with conventional training costing 25 €. Seventy-two educational sessions were therefore required to render tablet-PC training cost-effective.

## Discussion

In this randomized, controlled trial, tablet-PC education proved to be non-inferior in terms of improved immunosuppression-compliance compared to conventional education. Along with reduced variability in immunosuppression, significant improvements in patient knowledge were observed following further training. To our knowledge, this is the first study that studied tablet-PC education among lung-transplant recipients. Based on the described system, other educational themes after transplantation have been implemented.

### Education part

Ongoing patient education has become an established medical instrument, aiming to improve patients' knowledge about their disease and its treatment [24], [25]. Currently, structured programmes have been developed in a variety of chronic diseases (asthma, diabetes), augmenting medical therapy [26]–[29]. New concepts examining the role of e-learning have emerged in recent years and promises cost effectiveness [30]–[32]. In common with existing studies [30], [33]–[35], we could demonstrate non-inferiority of tablet-PC education in patients with demonstrating poor therapeutic adherence of their immunosuppression following lung-transplantation. Effectiveness of self-directed, computer-based education may be explained by increased attention that patients require whilst interacting with the device [30]. This repetition aids patients in retaining the information provided [30], [31]. Although e-education is a more standardized method than face-to-face education [31], face-to-face education offers a more individual teaching that can focus on individual problems of a given patient.

To maximize the cohort of patients capable of participating in interactive education, we chose an iPad®, due to its simpler handling when compared to standard laptop computers [36]. Additionally, the physical design of the iPad® afforded straightforward decontamination (deBac-app), which was considered advantageous in a potentially infectious and simultaneously infect-susceptible patient cohort.

### Cost

Computer-based, self-directed education helps reduce involvement of professional staff, which may result in economic benefits [31]. A cost-calculation revealed that e-education was however more expensive than conventional education with 45 vs. 25 € per session in this study. Beyond 72 patients however, e-education achieves cost-effectiveness and given that our center currently follows up almost 800 patients after lung-transplantation, the tablet-PC approach offers substantial savings, particularly given the continual expansion in educational themes being added to our repertoire. Positive effects of education (better immunosuppression drug levels) lead to lengthening intervals between drug measurements and reduced laboratory costs (24 € per measurement) as well as postal and the cost of calls to inform patients. If the number of required trough levels could be reduced by 50%, an annual saving of 300 € per patient would ensue.

### Immunosuppression

After multiple studies evaluating adherence, potential risk factors and the consequences of non-adherence [37]–[40], this is the first study investigating two strategies to improve immunosuppression after lung-transplantation.

### Aspects of patients medication adherence

Non-adherence can extend to other important aspects of patient cooperation e.g. communicating changes in health status between appointments and physical activity, which were not considered here. Stable therapeutic CNI drug levels result from patients' knowledge and discipline regarding medication consumption, correct intervals, drug metabolism and handling demanded by their inherent pharmacokinetics and –dynamics. All patients demonstrated good fundamental knowledge at inclusion in this trial (16/20 correct answers) and appeared to follow prescribed dosing of CNI. Adherence rating by physicians correlated with knowledge test results. Whereas patients with good adherence had better knowledge, there was no correlation of knowledge and improvement of therapeutic drug levels. Consequently, good knowledge about medication alone cannot prevent from non-therapeutic drug levels. We conclude that practical advice for daily handling of immunosuppressants were highly important in achieving good compliance. Patient evaluation of the education they received illustrated this aspect clearly. Evaluation of long-term improvements on patient survival following this intervention should be evaluated in future studies.

### Limitations

Larger trials testing for superiority are required to provide evidence of clinical benefit. Future studies are required to evaluate, whether education can help prevent acute and consecutively chronic lung allograft dysfunction [41].

## Conclusion

This randomised study proves positive effects of patient education on achieving improvements in therapeutic immunosuppression levels. Tablet-PC based education proved non-inferior to personal conventional education and may help physicians to improve effectiveness of education. Due to limitations in computer literacy and handling of electronic devices, specialist input was still required. Tablet-PC education now represents an integral component in our routine management of outpatients demonstrating poor immunosuppressive treatment control. Once established, the same equipment may be used for different aspects of patient education (e.g., therapy with azithromycine or bronchial stenting), adding greatly to their cost effectiveness.

## Supporting Information

Figure S1 **A** Patient with tablet-PC receiving education. **B – C** Screenshots from included video clips. **B** explanation of immunosuppression levels after intake and the consequences of missing or excessive intake. **C** patient with excellent drug levels and adherence describes tips. **D** demonstration of storage of immunosuppressive drugs in a car (influence of sunlight or cold). All individuals have given written informed consent, as outlined in the PLOS consent form, to publication of their photograph.
(PNG)

Table S1 **List of tips for patients after lung-transplantation.**
(DOC)

Checklist S1 **Consort checklist.**
(PDF)
