# Supplementary figures and images for: Conventional vs. Tablet Computer-Based Patient Education following Lung Transplantation – A Randomized Controlled Trial
Source: PLoS One. 2014 Mar 7;9(3):e90828. doi: 10.1371/journal.pone.0090828 (PMC3946627; doi:10.1371/journal.pone.0090828)

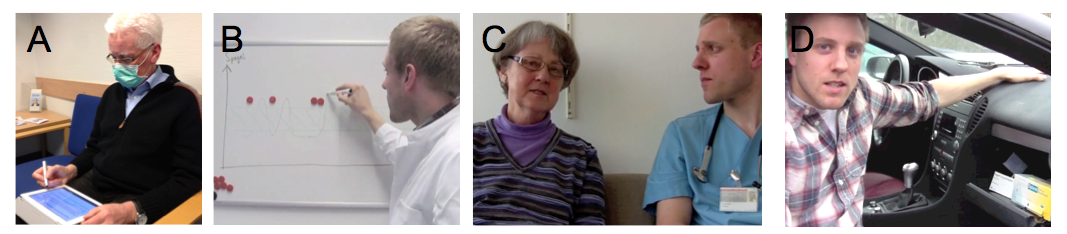

Supplement: Figure S1 — A Patient with tablet-PC receiving education. B – C Screenshots from included video clips. B explanation of immunosuppression levels after intake and the consequences of missing or excessive intake. C patient with excellent drug levels and adherence describes tips. D demonstration of storage of immunosuppressive drugs in a car (influence of sunlight or cold). All individuals have given written informed consent, as outlined in the PLOS consent form, to publication of their photograph. (PNG) [file pone.0090828.s001.png]
