# Supplementary material for: Conventional vs. Tablet Computer-Based Patient Education following Lung Transplantation – A Randomized Controlled Trial
Source: PLoS One. 2014 Mar 7;9(3):e90828. doi: 10.1371/journal.pone.0090828 (PMC3946627; doi:10.1371/journal.pone.0090828)
Supplement: Table S1 — List of tips for patients after lung-transplantation. (DOC) [file pone.0090828.s002.doc]

Supporting information 1

| **Education content (tips)** |
| --- |
| 1. CNI medication should remain in its blister, open the blister shortly before consumption (5 min). 2. Check dose of medication before intake. 3. Medication interval should be exactly 12 hours (720 min). 4. Time interval between meal and intake should be constant, ideally 1 hour before or 2 hours after. 5. Meal content (particularly fat) should be consistent, especially for breakfast and dinner. 6. In cases of severe vomiting, tacrolimus capsule should be placed under the tongue. 7. In case of regurgitation of complete CNI capsule, repeat the dose. In other cases of vomiting and regurgitation more than 1 hour after intake, no extra medication. 8. Forgotten medication should be taken as soon as possible. 12 hour-interval should be regarded. 9. Alarm (mobile telephone or clock) should remind every 12 hours. 10. On vacation, carry enough medication with you; medication to be carried in hand luggage. 11. No change of drug manufacturer. 12. Store at room temperature, shield medication from direct sunlight and low temperature. 13. Intake of medication before bronchoscopy is allowed. 14. Change medication and dosage, even of non-immunosuppressant medication, only following consultation with your physician to avoid interactions. 15. Call the lung-transplantation outpatient clinic if questions arise regarding medication. 16. Drug-levels should only be determined in a reference lab (MHH) and the next trough level should be checked after a minimum of 7 days. |
